# Successful Working Memory Processes and Cerebellum in an Elderly Sample: A Neuropsychological and fMRI Study

**DOI:** 10.1371/journal.pone.0131536

**Published:** 2015-07-01

**Authors:** Elkin O. Luis, Gonzalo Arrondo, Marta Vidorreta, Martin Martínez, Francis Loayza, María A. Fernández-Seara, María A. Pastor

**Affiliations:** 1 Neuroimaging Laboratory, Division of Neurosciences, Center for Applied Medical Research (CIMA), University of Navarra, Pamplona, Spain; 2 Department of Neurology, Clínica Universidad de Navarra, University of Navarra School of Medicine, Pamplona, Spain; 3 CIBERNED, Centro de Investigación Biomédica en Red de Enfermedades Neurodegenerativas, Instituto de Salud Carlos III, Madrid, Spain; 4 Department of Psychiatry, University of Cambridge, Cambridge, United Kingdom; University Medical Center Goettingen, GERMANY

## Abstract

**Background:**

Imaging studies help to understand the evolution of key cognitive processes related to aging, such as working memory (WM). This study aimed to test three hypotheses in older adults. First, that the brain activation pattern associated to WM processes in elderly during successful low load tasks is located in posterior sensory and associative areas; second, that the prefrontal and parietal cortex and basal ganglia should be more active during high-demand tasks; third, that cerebellar activations are related to high-demand cognitive tasks and have a specific lateralization depending on the condition.

**Methods:**

We used a neuropsychological assessment with functional magnetic resonance imaging and a core N-back paradigm design that was maintained across the combination of four conditions of stimuli and two memory loads in a sample of twenty elderly subjects.

**Results:**

During low-loads, activations were located in the visual ventral network. In high loads, there was an involvement of the basal ganglia and cerebellum in addition to the frontal and parietal cortices. Moreover, we detected an executive control role of the cerebellum in a relatively symmetric fronto-parietal network. Nevertheless, this network showed a predominantly left lateralization in parietal regions associated presumably with an overuse of verbal storage strategies. The differential activations between conditions were stimuli-dependent and were located in sensory areas.

**Conclusion:**

Successful WM processes in the elderly population are accompanied by an activation pattern that involves cerebellar regions working together with a fronto-parietal network.

## Introduction

With advancing age, most adults report difficulties in generating strategies to maintain and handle information in rapidly changing social contexts. One of the most studied cognitive processes in the neuroscience of aging is working memory (WM), which has been defined as the process that maintains limited information in the mind during short periods of time [1]. An accepted model proposes that WM processes are carried out by specific subsystems: the Phonological Loop (PL), the Visuo-spatial Sketchpad (VS) and the Episodic Buffer (EB). Both the PL and VS have a passive storage system, and a loop-strategy of active maintenance of information [2]. A fourth construct called the Central Executive (CE) works over the slave subsystems through the orientation, focus and division of attentional resources [3,4].

Currently the roles of the basal ganglia (BG) and the cerebellum in WM-processing are better recognised. McNab et al. [5] demonstrated with functional magnetic resonance imaging (fMRI) that BG activity was a predictor of unnecessary storage and played a role as a filter of irrelevant information for WM. Another fMRI study conducted by Ziemus et al., [6] using a 2-back paradigm showed that cerebellar lesions affect the functioning of the fronto-parietal network, which is involved in WM.

Bower [7] regards the cerebellum as a perception facilitator through the coordination of sensory information acquisition. In WM, cerebellar function has been proposed to depend on stimuli conditions, memory load or task type [8]. The cerebellar function, and particularly the function of VI/Crus I and VIIB lobules, might not be simply related to maintaining the memory footprint, but also to executive control [9]. Other studies have shown that right VI and Crus I are involved during subvocal rehearsal processes (SRP) and lobules VIIB and VIII during the phonological store (PS) [10,11]. Whereas a left predominance for visual input conditions has been reported [12], other investigations suggest that the cerebellum might serve as an interface between the two components of the phonological loop (SRP and PS), comparing the output of SRP with previously stored content [10,13,14].

Previous studies have shown a cognitive decline associated with age in tasks supported by the frontal lobes, such as WM, priming and text processing [4]. In addition, it has been proposed that age-related changes are the result of a weakening of inhibitory processes in WM (the inhibition deficit hypothesis). Recent neuroimaging studies have identified several changes in brain activation patterns during WM tasks due to aging. Such studies have demonstrated that older adults exhibit greater activation in prefrontal regions coupled with lower activations in posterior brain regions when performing WM tasks, supporting the sensory deficit hypothesis. It has also been reported that older adults during WM tasks show greater vulnerability to distractions that weaken the memory trace and an age-associated decline in their performance on WM neuropsychological tests [15,16].

Several studies have reported that aging involves a differential impairment of visuospatial and language functions [17]. Studies that have compared different stimuli conditions or various memory loads in elderly participants have found differences between visual and auditory conditions in areas responsible for sensory processing [18]. On the other hand, an age-related overactivation of frontal areas during the processing of WM tasks has been observed as condition-independent, concurrently with a decrease of activation in sensory cortices [10,19,20]. However, it has been suggested that the use of different tasks and stimuli may lead to different functional activation maps [10,18].

In this work, we study WM in a sample of neuropsychologically healthy elders. The study aimed to test three hypotheses. First, that the pattern of brain activation during low-load WM-task execution in a sample of successful WM-aging involves posterior sensory and associative areas. Second, that the dorsolateral prefrontal cortex (DLPFC), parietal cortex and GB activations, should be greater in cognitively demanding tasks. Third, that cerebellar activations will be greater in cognitively demanding tasks and that they will show a condition specific lateralization. In summary, our objective is to characterize WM function in an adult population without neuropsychological alterations using an N-back paradigm and comparing different conditions of stimuli and loads.

## Materials and Methods

### Subjects

Twenty right-handed volunteers (half of whom were men) of age ranging from 58 to 66 years old (mean 62.2 and standard deviation (SD) 4.9) were recruited after screening an initial pool of 42 volunteers belonging to the senior program at the University of Navarra. The Mini–mental state examination MMSE [21] **(29.2 ± 1.2)**, Alzheimer's Disease Assessment Scale-cognitive ADAS-Cog [22] **(3.85 ± 2.4)** and the 30 item Geriatric Depression Scale GDS [23] **(5.35 ± 3.5)** were used in the screening phase (cutoffs 28, 12 and 9, respectively), during which 22 subjects were discarded due to their results falling below the pre-specified thresholds. The final sample of participants reported an average of 12.4 (SD 2.3) years of education. The study was approved by the Ethics Committee of the University of Navarra and participants signed a written informed consent prior to the study.

### Design and procedure

#### Neuropsychological Assessment

We determined cognitive status in our sample using part of the Wechsler Intelligence Scale WAIS-III (Seisdedos et al., 2001), with specific subtests comprising *Vocabulary*, *Comprehension*, *Similarities*, *Digits*, *Information* and *Letters and Numbers*. Four non-standardized tests assessed the psychological effects of the multicomponent theory of WM, namely *the word length*, *articulatory suppression*, *the phonological similarity effect and the irrelevant sound effects*. Tests were carried out as reported previously [1]. Further tests were the Stroop [24] and the visual patterns test (VPT) [25]. Finally, we determined storage capacity (verbal span and visuospatial span) from the digits subtest and the VPT.

All comparisons between conditions (for both WAIS-III as for the four non-standardized tests) were carried out using the Wilcoxon signed-rank test (W-test from now on) [26]. Behavioural comparisons were carried out with IBM SPSS and a statistical p value below 0.05 was considered significant.

#### *fMRI* paradigm

The paradigm consisted of eight N-back tasks, differentiated by memory load (high or low) and stimulation condition (visuo-phonological (VPh), audio-phonological (APh), visual (V) and spatial (S)). The study was divided into two scanning sessions: during the first one, participants carried out VPh and APh tasks, while they underwent V and S during the second. The phonological and visuo-spatial paradigms were modified and adapted from [27]. For the verbal tasks (VPh and APh), 18 consonants were used, whereas for the visual (V) and spatial (S) tasks, 18 characters of the Mandarin alphabet were employed. The VPh, APh and V tasks consisted in a typical N-back procedure [28]. Conversely, in the S task the same stimuli that in the V condition were used. One of these stimuli was placed in one of four possible positions and the participant had to indicate if the position of the stimulus presented as the response matched that of the target. Participants had to ignore stimulus and respond only to position.

**Fig 1:**
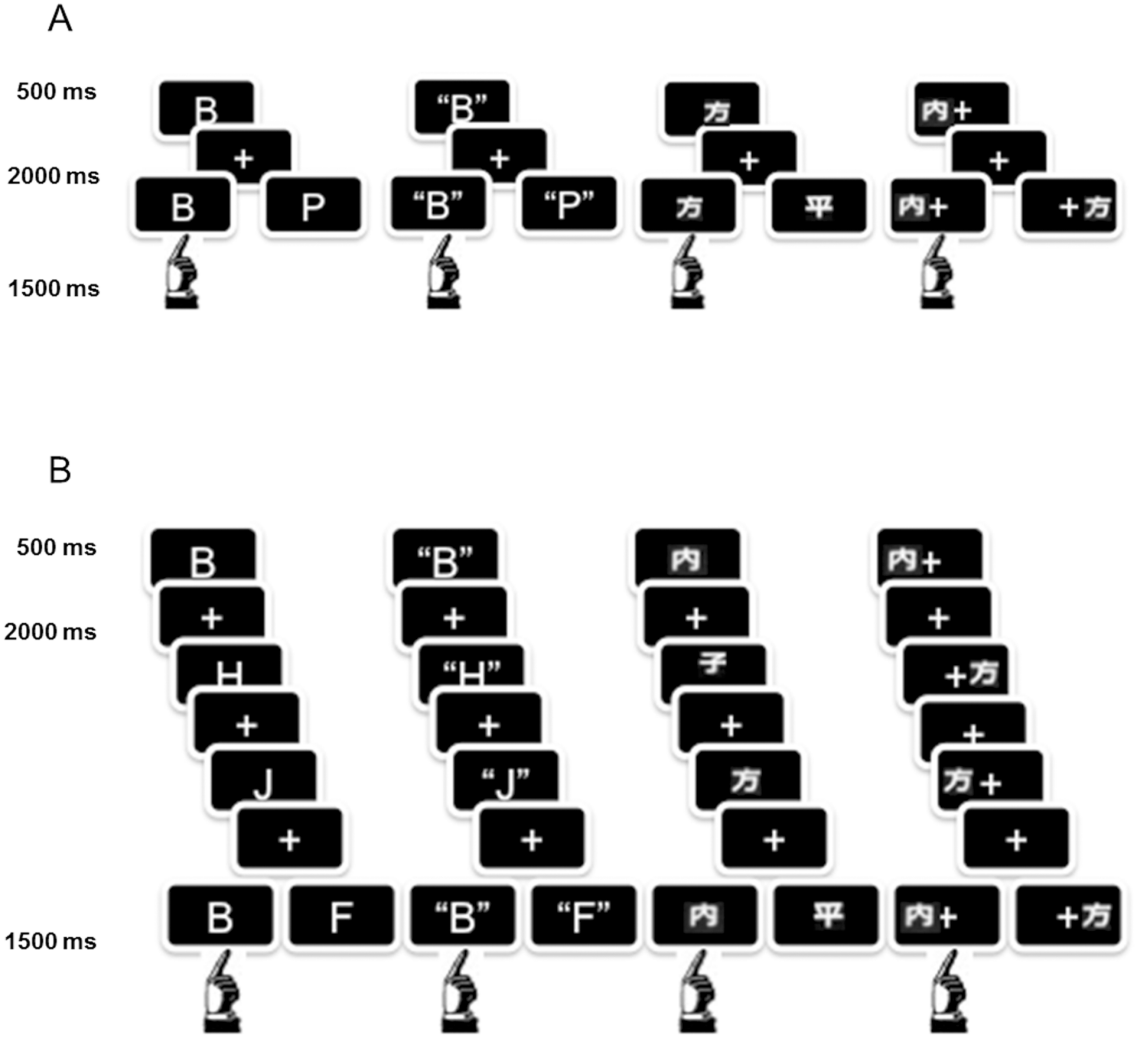
Scheme of the presentation in the four stimulus conditions. Above: low memory load. Below: high memory load. A: VPh, B: APh, C: V and D: S. In each case if the left answer was presented the participant would have to answer “match” whereas if the answer shown was the one to the right the subject would have to respond “mismatch” with the button box.

Regarding the two memory loads, for the remainder of the article high-load conditions will be abbreviated with the prefix H and low-load with L (e.g. HPV for the high load visuo-phonological verbal condition, LV for the low-load verbal condition). In the low memory load paradigms, a 1-back task was employed (block duration = 16 s). For the high load paradigms, a 3-back task (block duration = 36 s) was used. The time of stimulus presentation was 500 ms with an interval between stimuli (ISI) of 2000 ms. Subjects were instructed to respond true (match) or false (mismatch) by pressing a different button in a response box. Answers matched the cue in half of the events and the order of matches and mismatches was pseudo-randomized (Fig 1).

A control task was designed for each condition. Only stimuli X or XX in the case VPh tasks or ו or װ (from the Mandarin alphabet) for the visual tasks, were presented. Conversely, for APh tasks letters “X” or “XX” were named orally. On the other hand, for the S control the same stimuli as for the task were used, but stimuli only appeared either on the right (ו) or left (װ) of the fixation crosses. In all cases, the participants were requested to press the button corresponding to the correct answer.

Within each scanning run 9 control blocks alternated with 9 task blocks. Blocks consisted of 4 events in each condition so 36 tasks and 36 control events were presented per run. Low load runs of each condition always preceded high load runs.

#### Scanning protocol

Imaging was performed using a 3T scanner (Trio TIM, Siemens AG, Erlangen, Germany) equipped with a 12-channel head array coil. The functional images sensitive to blood oxygenation level dependent contrast (BOLD) were acquired using a T2*-weighted echoplanar imaging sequence and the anatomical images were acquired with an MPRAGE sequence.

#### Image pre-processing

Image processing and statistical analyses were carried out using Statistical Parametric Mapping (SPM8) (http://www.fil.ion.ucl.ac.uk/spm/). The structural image was segmented using the “New Segment” tool of SPM while functional images of each subject were realigned to the mean image of their corresponding session, co-registered to the anatomical dataset and skull-stripped, then spatially normalized to the Montreal Neurological Institute (MNI) reference brain template and finally spatially smoothed with an 8-mm isotropic Gaussian filter.

#### Performance analysis

In order to evaluate subject's performance during the functional tasks, we measured the reaction time of the correct responses (mean RTcr) and the proportion of correct responses. These data were obtained for both tasks as well as for control tasks. The effect of load and condition on the proportion of correct responses and RTcr were analysed with two repeated-measures ANOVA and Bonferroni post-hoc correction.

#### Statistical analysis of the images

Imaging data corresponding to the correct answers were analyzed first at the subject-level, and then at the group-level. At the subject-level, the time series of the eight conditions were analyzed within the same first-level design matrix. In an initial approach, we compared the task and control events from both the low- and high-load presentations obtaining one contrast for each condition. These contrasts were then entered into a conjunction analysis. Subsequently, we compared task and control events within each condition and entered these 8 contrasts per subject into a second level ANOVA in order to search for activation differences due to input condition and load. Additionally, task-control contrasts were included in a PPI analysis to evaluate effective connectivity within each condition.

First-level analyses were modelled with a canonical double gamma hemodynamic response function (HRF) [29]. Second level activations were evaluated at p < 0.05, using a familywise error rate (FWE) correction for multiple comparisons at the cluster level after a primary threshold of p < 0.001.

#### Activation within each condition

We analysed each condition and load independently by creating 8 separate one-sample t-tests, in which we entered each subject’s task-control contrast (one per condition). Age, education and verbal IQ were introduced as covariates in the phonological condition tests, while age, years of education and cubes score (WAIS-III) were included in the visuospatial tasks.

#### Differential activations between conditions and loads

To determine the differential activation between memory load levels and conditions, we implemented a 2 x 4 ANOVA including the task-control contrasts as input images. We modelled the ANOVA as a flexible factorial which had subjects, load, condition and load x condition as factors in the design matrix. F-tests were used to obtain the SPMs of the main effects and interactions. Afterwards, post-hoc t-tests were carried out.

#### Exploratory analyses

With the aim of characterizing WM function in the elderly according to Baddeley´s model and identifying the role of subcortical areas in WM, we performed two analyses.

First, in order to identify the regions involved in processes common to all the tasks, we carried out a “masked conjunction” analysis of the four sensory conditions. A task vs. control contrast was obtained at the subject-level combining low- and high-load scans for each condition. The individual t-contrasts were included in a separate group model for each condition and we then thresholded the group t-contrasts using our predetermined statistical correction and converted the SPMs into binary images. Finally, we overlapped these masks to find common areas. We thus obtained a qualitative map ranging from 1 (no overlap) to 4 (maximum overlap). The logic of this approach is highly analogous to a null hypothesis conjunction analysis for the maximum overlap and to the intermediate and global null hypothesis for all other overlaps [30].

The second was an effective connectivity analysis that assesses whether activity in one brain region can be explained by an interaction between a cognitive process and activity in another part of the brain. We used the psycho-physiological interactions (PPI) method [29] to estimate effective connectivity changes for the Ventro lateral anterior thalamic nucleus (13, -4, 10); the Globus Pallidus (20, 0, 12) and the Cerebellum (Lobule VI: left -29, -57, -30; right 26, -57, -27; Lobule VIIIA and Crus I: 38, -58, -30; Vermal Lobule IX: 1, -59, -29), where the neural activity increased during low and high loads.

Seeds for the PPI analysis were based on the maximal overlap at the subcortical level in the conjunction analysis (i.e. the common subcortical areas of activation between the four conditions). For every condition, we found the highest activation peak from its task-control contrast within a mask equal to each of the clusters of maximal overlap in the conjunction analysis. We then drew an 8mm radius spherical volume of interest (VOI) centred on this peak for each subject and WM task. The data are fully available without restriction in the Neuroimaging Laboratory server (Division of Neurosciences, Center for Applied Medical Research (CIMA). University of Navarra, Pamplona, Spain), for request, however the support information provided the resources about data processing and data analysis.

## Results

### Neuropsychological tests

There was no global cognitive impairment (Table 1) or mood disorders in our sample. Additionally, all participants showed WM-effects reported for multi-component model of WM (Table 2). This may suggest the optimal state of the WM-processes in our sample.

**Table 1:** Descriptive Scalar Scores on Subtests of the WAIS-III.

WAIS-III	
Test*	Median ± IQR
**Vocabulary**	11.5 ± 2
**Similarities**	9.0 ± 3
**Block design**	12.5 ± 3
**Arithmetic**	11.0 ±1
**Digit**	12.5 ±3
**Digit forward span**	6.0 ± 2
**Digit backward span**	5.0 ± 1
**Information**	10.5 ±2
**Comprehension**	12.0 ±2
**Letters and numbers**	9.5 ±2
**IQ verbal memory**	107 ±8
**Comprehension verbal index**	102 ±8
**IQ working memory**	106 ±7

* Raw scores of the WM-effects;

**Table 2:** Descriptive data of the Stroop test, Non-standardized tests and Visual Patterns test.

Test	Subtest	Median±IQR	p effect
**Articulatory suppression effect** *	Test with articulatory suppression vs. Test without articulatory suppression	37.5 ±10 vs. 50.0 ± 6.0	<0.001
**Word length effect** *	Short words vs. Long words	37.0 ± 3.0 vs. 34.0 ± 6.0	<0.001
**Phonological similarity effect** *	Phonological similarity vs. Phonological difference	27.0 ± 5.0 vs. 36.0 ± 6.0	<0.001
**Irrelevant sound effect** *	Test with irrelevant sound vs. Test without irrelevant sound effects	26 ± 1.7 vs. 42 ± 3.2	<0.0001
**Visual patterns test** *	Number of successful matrices	10 ± 4	---
	Span	6.0 ± 2	---
**Stroop** **	Words	11.5 ±4.0	---
	Colour	13.0 ±4.0	---
	Word / Colour	15.0 ±6.0	---

* Raw scores of the WM-effects;

** Scalar scores of Stroop test.

### *fMRI* study

The effects of WM load and condition on the proportion of correct responses were not significant, although overall lower scores were observed in high-load vs. low-load tasks. In the control task, we observed the best scores on phonological paradigms. The interaction between condition and load in control tasks corroborated best scores for phonological paradigms, specifically in low load (S1 Table).

Condition influenced RT: post hoc comparisons showed longer *RTcr* in the visuospatial conditions compared to the phonological conditions and between these, longer *RTcr* were observed in the VPh condition. For control tasks, we observed an interaction between condition and load that significantly showed longer *RTcr* in both loads for VPh, V and S vs. APh, VPh vs. S and V vs. S (Table A in S2 Table and Table B in S2 Table).

#### Functional activation in the WM tasks

We found a consistent fronto-parietal pattern of activations in all of the WM tasks, independently of the input condition or WM load. Areas activated in most contrasts included middle and inferior occipital gyri, superior and inferior parietal gyri, middle and inferior temporal gyri, precentral gyrus, supplementary motor area (SMA), anterior, middle and posterior cingulate cortex (ACC, MCC and PCC), DLPFC and ventrolateral prefrontal cortex (VLPFC), and cerebellum (lobule VI, VIIA and Crus I).

Qualitative differences between conditions included an increased recruitment of subcortical and cerebellum areas in visuospatial tasks compared to phonological paradigms. Additionally, with greater memory loads, we observed bigger clusters of activation in subcortical, cerebellum and occipital regions (S1 Fig).

#### Differential activations between loads and conditions

**Fig 2:**
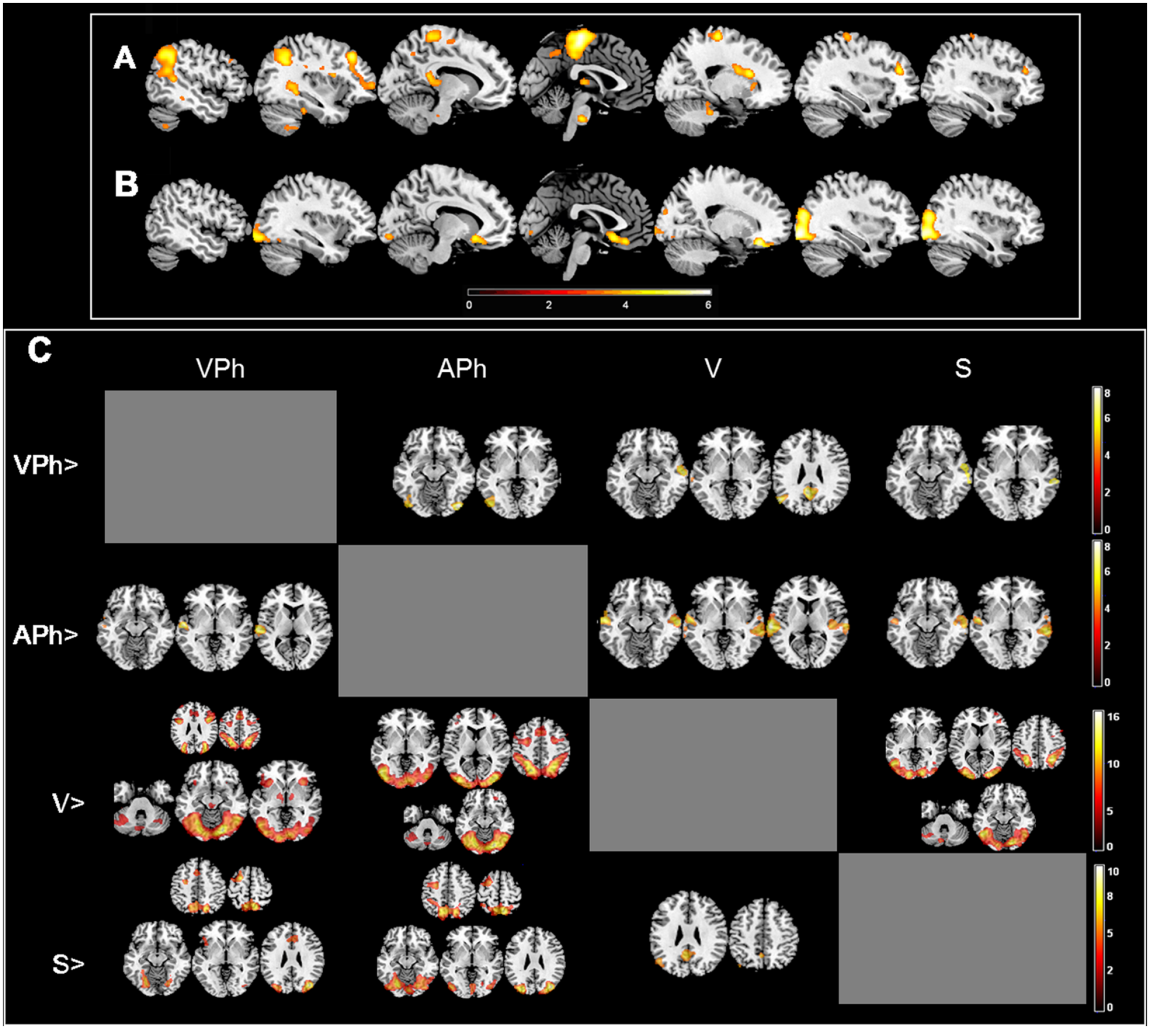
Effect of WM memory load in sagital plane and differences between conditions in axial plane. A. Low > High loads, B. High > Low loads and C. Factorial design results, (p < 0.05 FWE cluster-wise corrected p = 0.001).

Areas more active in low loads vs. high loads were the bilateral middle and inferior occipital gyri, temporal and orbitofrontal regions, whereas differential activations of high load were bilaterally located in the superior and middle temporal gyri as well as the thalamus (Pulvinar nucleus) and caudate nucleus. Furthermore, there was an increased activation of the right SMA and ACC in high memory load tasks vs low loads. In the left hemisphere, we observed activations in areas such as the paracentral lobule, hippocampus, inferior parietal, DLPFC and orbital gyri and cerebellum (left VIIB and right VI lobules) (Fig 2A and 2B and Table A in S3 Table and Table B in S3 Table).

Areas showing a main effect of condition were located in the superior and medial temporal gyri, medial and inferior occipital gyri, fusiform, lingual and inferior parietal gyri, ACC, and inferior and middle frontal gyri (Fig 2C).

Specifically the VPh vs. APh contrast showed differential areas of activation in the inferior occipital and temporal gyri and cerebellum, while in the VPh vs. V contrast we found differential activation in bilateral middle temporal and superior temporal gyri, angular gyrus and PCC. Finally, VPh had more activity than S only in the middle temporal gyrus (S4 Table).

APh showed a stable pattern of activations as it had an increased activity in the superior and middle temporal gyri when compared to any of the other conditions. V also showed similar SPMs across comparisons; areas more active in V than in the remaining conditions were the cerebellum, most of the occipital lobe and frontal areas such as the superior, medial and inferior frontal gyri (S5 Table).

Regarding V vs. both phonological versions, we found a similar pattern in areas of the bilateral middle occipital gyrus, ACC, inferior and middle frontal gyri. However, in V vs. VPh differential activations were observed in the insula lobe, SMA, cerebellum, pallidum, and caudate nucleus. Additionally to the pattern described before for the V vs. APh contrast, we also found differences in the precentral gyrus, SMA, ACC, insula and lingual gyrus. The comparison between V and S showed activation in the inferior temporal, parietal and frontal gyri and lingual gyrus (Table A in S6 Table and Table B in S6 Table).

On the other hand, when S and the phonological paradigms were compared we observed a similar occipito-parieto-frontal network of clusters in both cases, whereas some temporal and parietal areas and the posterior cingulate cortex were more active in V than S (Table A in S7 Table and Table B in S7 Table).

We found an interaction between load and condition driven by differences among conditions in low load. Specifically, the significant post-hoc contrasts were LV vs. LAPh, LV vs. LVPh and LV vs. LS with differences located in the right middle occipital gyrus. In addition to this area, the first and third contrast activated the right inferior occipital gyrus and cuneus, and the second the right calcarine gyrus.

#### Exploratory analyses

**Fig 3:**
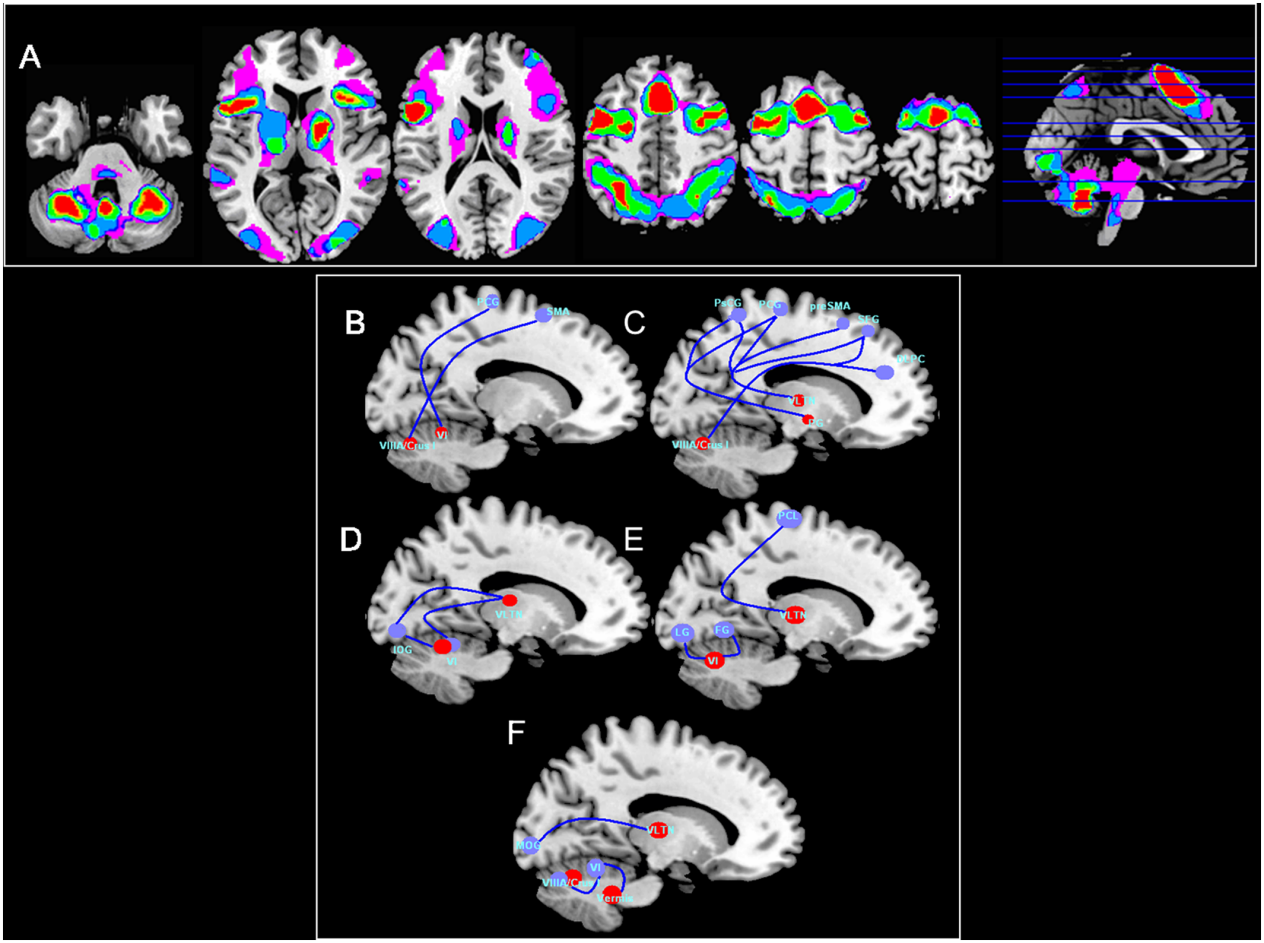
Areas of conjoint activation across the different WM stimulation conditions and PPI analyses. A. Red: areas active in the 4 conditions. Green: 3 conditions. Blue: 2 conditions. Violet: 1 condition and PPI conditions and loads. B: High visual phonological task. C: High auditory phonological task. D and E: Low and High loads in visual task, receptivity. F: High load spatial task. (p < 0.05 FWE cluster-wise corrected on voxels with p < 0.001).

We performed a conjunction analysis for the stimulus conditions to identify regions involved in common processes to all tasks. Activation was common to all conditions in areas such as the inferior frontal gyrus, ACC, insula, SMA, precentral and parietal gyri and at the subcortical level in the right thalamus (Ventral lateral anterior nucleus VLATN), pallidum and cerebellum (VI, VIIIA/Crus I lobule and Vermis). The overall pattern of activations can be described as highly symmetrical. The recruitment of the primary and secondary visual areas was common to all three tasks with visual input (VPh, V and S) and especially to V and S. Moreover, a fronto-parietal pattern of activations was greater in visuospatial than phonological paradigms. Additionally, we observed a bilateral activation of the thalamus in APh, V and S. Finally, phonological paradigms shared activations in parieto-temporal areas (Fig 3A).

Subsequently, we conducted several PPI analyses for each of the 8 conditions with seeds located in subcortical and cerebellum regions common to all WM tasks in the conjunction analysis. For low load conditions, only the V task showed significant connectivity. Particularly for this task, VLATN and left occipital lobe interacted with the cerebellum (Crus I lobe). Besides the left VI cerebellar lobe presented an interaction with left Crus I lobe and left inferior occipital lobe (Fig 3 and S8 Table). We observed a connectivity pattern in the V paradigm between the right VLATN and bilateral paracentral gyri. Other regions such as the lingual and fusiform gyri were associated with the right cerebellum VIIIA and Crus I (Fig 3E and S8 Table).

During high WM memory load, the VPh task showed an increase in connectivity between the right cerebellum VIIIA/Crus I lobes and the bilateral SMA. In addition, the right VI cerebellar lobe showed connectivity with the left precentral gyrus Regarding the APh connectivity, we observed that this condition was more robust compared with other conditions: a predominantly interhemispheric integration was observed between the right VLATN with bilateral pre SMA, left postcentral and precentral gyri, right globus pallidus with left postcentral and precentral gyri. Finally, the right cerebellum also interacted with the superior medial frontal gyrus, left insula lobe and bilateral DLPFC (Fig 3C and S9 Table).

Lastly, the right VLATN presented connectivity with the medial occipital gyrus in S task. In addition, right cerebellum interacted with right VI lobe and contralateral VIIA/Crus I and the vermal lobule IX with right VI lobe (Fig 3F and S10 Table).

## Discussion

In this work, we have studied the neural substrates of WM in a group of healthy elders using an N-back paradigm with four types of stimulus conditions and two memory loads. We found that successful WM processes in this population are accompanied by an activation pattern that involves not only a fronto-parietal network but also cerebellar regions. In particular, the cerebellum was distinctly activated during the tasks that required higher executive demand. This activation pattern was condition independent. Together with the cerebellum, high-load tasks involved areas such as the DLPFC, parietal cortex and BG, which might support WM functions (i.e. information manipulation, inhibition and updating). On the other hand, an occipito-orbitofrontal activation pattern compatible with the ventral visual recognition network was active during low-load tasks. These results indicate an increased subcortical-cortical activation and connectivity pattern with increasing memory loads, suggesting a prominent role of the cerebellum in successful WM processing in the elderly.

### Greater BG and cerebellum recruitment on high-memory load

Our results on high memory load paradigms demonstrate the involvement of the caudate nucleus, the pulvinar thalamic nucleus (PN), cerebellum and hippocampus in addition to the frontal and parietal cortex. The caudate nucleus belongs to the associative circuit [31] and its role in switching between cognitive strategies and in the generation of goal-directed behaviour has been defined in fMRI studies [32–34]. Regarding the activation of the PN, it participates in the encoding of visual information; assigning visual attention and filtering irrelevant information in WM-tasks [35]. In the present study, cerebellar activity could be associated with the modulation of executive resources. Cerebellar recruitment was greater with increased stimulation complexity. Increased activation was present during all high-load tasks. Independently of load, activation was also greater during the visual tasks, which are the most complex presentations in our paradigm [6]. This is consistent with the results of two meta-analyses that have reported the involvement of cerebellar regions in executive control functions [9,36,37]. A recent consensus paper on the role of the cerebellum in perceptual processes states precisely that “the cerebellum is invoked in proportion to the need for sensory vigilance” [38], a prediction that is observed in our results, where cerebellar activation is greater when processing more complex tasks.

Another relevant finding was the involvement of the hippocampus in high load tasks. This result may be explained by the use of long-term memory to generate a more elaborate representation of the Chinese graphs or Spanish letters as a strategy for their maintenance in WM [39–41]. In this sense, the hippocampus plays a central role in the processing of complex elements and in the integration of long-term memories with short-time storage. These results support the notion that some of its sub-regions are the neural substrate of the Episodic Buffer [42].

### Greater sensory and associative recruitments on low-memory load tasks

Results corroborate the activation of sensory or association areas but they also show activation of the ventral visual recognition network [43], which is associated with object recognition and form representation. In addition, our sample presented a predominantly posterior activation pattern during low memory load. This situation is opposite to the posterior-anterior shift in aging pattern (PASA), which is defined as a reduction in occipital activity coupled with increased frontal activity [18, 44, 45].

### Differences between conditions

Differential activations among conditions were mainly restricted to areas responsible for the sensory processing of the stimuli used in each task. These results are in agreement with the work done by Fakhti et al., (2013) using the Sternberg paradigm. These authors found differences between tasks, which they attributed to the type of stimuli used. When we compared responses for phonological stimuli delivered through two input conditions (VPh vs. APh) the difference was significant in areas involved in the visual processing of letters, with similar activated regions to those previously reported [18,46]. We found differential activation between VPh and V paradigms in bilateral hippocampal areas, the PCC and angular gyrus, areas involved in verbal memory [47] and in the recognition of familiar words, objects, and places [48].

Comparisons between APh and other conditions, similarly to previous works, showed activation in the superior temporal gyrus, which is responsible for auditory recognition [10,18]. When confronting V and VPh/APh conditions, we observed similar occipito-frontal activation patterns. The bilateral pallidum and right caudate nucleus were more active in the V versus VPh contrast. This may be explained by a greater use of inhibitory strategies of irrelevant information in the visual task than in the visual-phonological task [49]. The comparison between the V and S conditions showed activation on inferior frontal, parietal and temporal areas. This increased recruitment in visual association areas underlines the possible utilization of associative strategies to maintain the memory trace in this condition. Finally, occipital and pre-cuneal activations were observed when comparing the S condition to the other paradigms. These regions are often reported as the dorsal visual recognition pathway [43].

In line with previous studies [50], and contrary to those reported by [10–12], we did not find cerebellar activations in phonological and spatial contrasts. This may be due to executive control dominating the phonological loop and visuospatial sketchpad, which, in turn, could be cancelling activation in those regions. Other possible explanation for these findings may relate to the cerebellum´s role in the control of sensory-data acquisition [7,38,51,52]. Sensory-data acquisition is present in all tasks and hence, the overall result may be a general cancellation of cerebellar activation. However, we observed a bilateral increase in BOLD activation in the VI, VIIA/B, Crus I and X cerebellar lobes, globus pallidus and caudate nucleus in the V condition compared to both phonological versions and S tasks. This is probably due to the higher difficulty of the V condition. Alternatively, it could be attributed to an increase of the vigilance processes. Such an increase would be induced by an interaction between task type and participants, as reported in studies carried out by Bower et al., [7]. In other words, older participants may require greater executive control strategies in the more difficult condition [12]. Findings support cerebellar involvement in sensory information control and executive processes [53].

### Common fronto-parietal cerebellar activation pattern

With the aim of characterizing the neural substrates of successful working memory processes in aging, we performed a conjunction analysis, in which we found a similar functional network involved in all forms of WM independently of the input condition or load. Additionally the cerebellum was active in all conditions. Studies with monkeys have reported reciprocal connections between Crus I and DLPFC [54]. Functional imaging studies in humans have reported that Crus I [55] and VIIIA [37] are involved in WM tasks. In resting-state functional connectivity studies there is evidence of the participation of Crus I and VI lobules together with frontal and parietal regions in the executive control network [44]. This network has been typically described as fronto-parietal. However, other studies have also pointed out the crucial role of the cerebellum in WM tasks [10]. Our results, combined with these previous findings, support a new concept of the WM network as a fronto-parieto-cerebellar network. Studies in patients with isolated cerebellar infarcts that presented WM, verbal fluency and attention impairments showed angular gyri and inferior parietal lobule overactivation during a 2-back WM task. These overactivations were suggested to be a compensation process for the altered functioning of the cerebellum [6]. Lobule VII, interconnected with the prefrontal cortex in non-human primates [45] has been found activated using the PASAT in consolidated skills of the maintenance and manipulation of information in verbal working memory [56].

The activation pattern obtained was clearly symmetrical and highly significant in the case of the frontal and cerebellar regions, in contrast with the predominantly left activation in parietal regions. A possible explanation is that left hemisphere dominance could be related to the implementation of verbal storage strategies in all conditions. It is interesting to note that humans’ prefrontal cortex has expanded more than the primary motor cortex and similar expansions are observed in the cerebellar cortical areas to which the prefrontal cortex is connected. Compared to non-human primates Crus I and Crus II demonstrated the largest differences between species being greater in humans followed by lobule VIIb y VIIIa [57].

### Connectivity

Cerebellar regions played an important role of in all conditions [58]. In both high load phonological conditions, we found cerebellum-cerebral connectivity between VIIIA-Crus I lobes and superior frontal regions. This connectivity can be associated with monitoring and manipulation processes in WM task [6,51,59]. APh showed a greater activation between DLPFC and cerebellum probably due to a greater demand of executive resources. Additionally, the connectivity found between cerebellum and globus pallidus in HAPh could be associated with reward-related processes that support WM, in a condition that shows the best performances together with the lowest response times [60]. Future studies should try to address how the interaction between the cerebellum and the BG influence WM processes [61].

The low memory load in the V condition triggered a connectivity pattern between VLATN and cerebellum with striate cortex and Crus I lobe [51]. The high load V showed cerebellum connectivity with extra-striatal regions and the S condition showed intra-cerebellar connectivity between VIIIA/Crus I lobes and VI lobe and cortico-cerebellar connections between VLATN with occipital regions. These results support the idea that an intra-cerebellum network is involved in the maintenance of complex information in WM or when cognitive demands increase in the task [10,11].

## Conclusions

Subcortical-cortical activations increase with greater memory loads in the elderly population. Our approach, centered on differences between memory loads, has not only demonstrated involvement of the frontal and parietal cortices but also activations of BG, PN and cerebellum in high memory loads. Moreover, we expected activation in sensory and association areas in low memory loads in neuropsychologically healthy elderly individuals. However, we found activations in the visual ventral network associated with the maintenance of short-term stimuli.

Cerebellum activations showed an important role in a relatively symmetric fronto-parieto-cerebellar network. This network was involved in all forms of WM independently of the input condition or load. Our elderly sample had a predominantly left activation in the parietal regions compared to the frontal ones.

Comparisons across conditions indicate an independent condition-activation in areas responsible for sensory processing; whereas the activation of multimodal association and the hippocampal regions are probably the result of using association strategies for the maintenance of the memory trace. Similarly, the recruitment of BG in the V condition may be related to the increased use of upgrade and inhibitory distractor strategies.

The connectivity analyses between superior frontal regions and cerebellum in the phonological conditions are probably involved in monitoring and manipulation processes. Finally, the cerebellum arises as an important structure with a prominent role in increasing cognitive demands and in the maintenance of complex information.

## Supporting Information

S1 Fig Activation maps by condition and memory load in sagital plane. VPh: visual phonological version; APh: Auditory phonological version; V: visual; S: spatial; L: Low and H: High. The color scale represents T-statistic values. (p < 0.05 FWE cluster-wise corrected on voxels with p < 0.001).
(TIF)

S1 File Introductory Notes. (PDF)

S1 Table Data correct responses descriptive for each condition and load. (PDF)

S2 Table Reaction time differences between conditions (correct responses only). (PDF)

S3 Table Differences between load activations analysis. (PDF)

S4 Table Differences between VPh and other conditions. (PDF)

S5 Table Differences between APh and other conditions. (PDF)

S6 Table Differences between V and other conditions. (PDF)

S7 Table Differences between S and other conditions. (PDF)

S8 Table PPI for low and high loads Visual. (PDF)

S9 Table PPI for high load VPh and APh. (PDF)

S10 Table PPI for high load S. (PDF)
