# Supplementary figures and images for: Successful Working Memory Processes and Cerebellum in an Elderly Sample: A Neuropsychological and fMRI Study
Source: PLoS One. 2015 Jul 1;10(7):e0131536. doi: 10.1371/journal.pone.0131536 (PMC4488500; doi:10.1371/journal.pone.0131536)

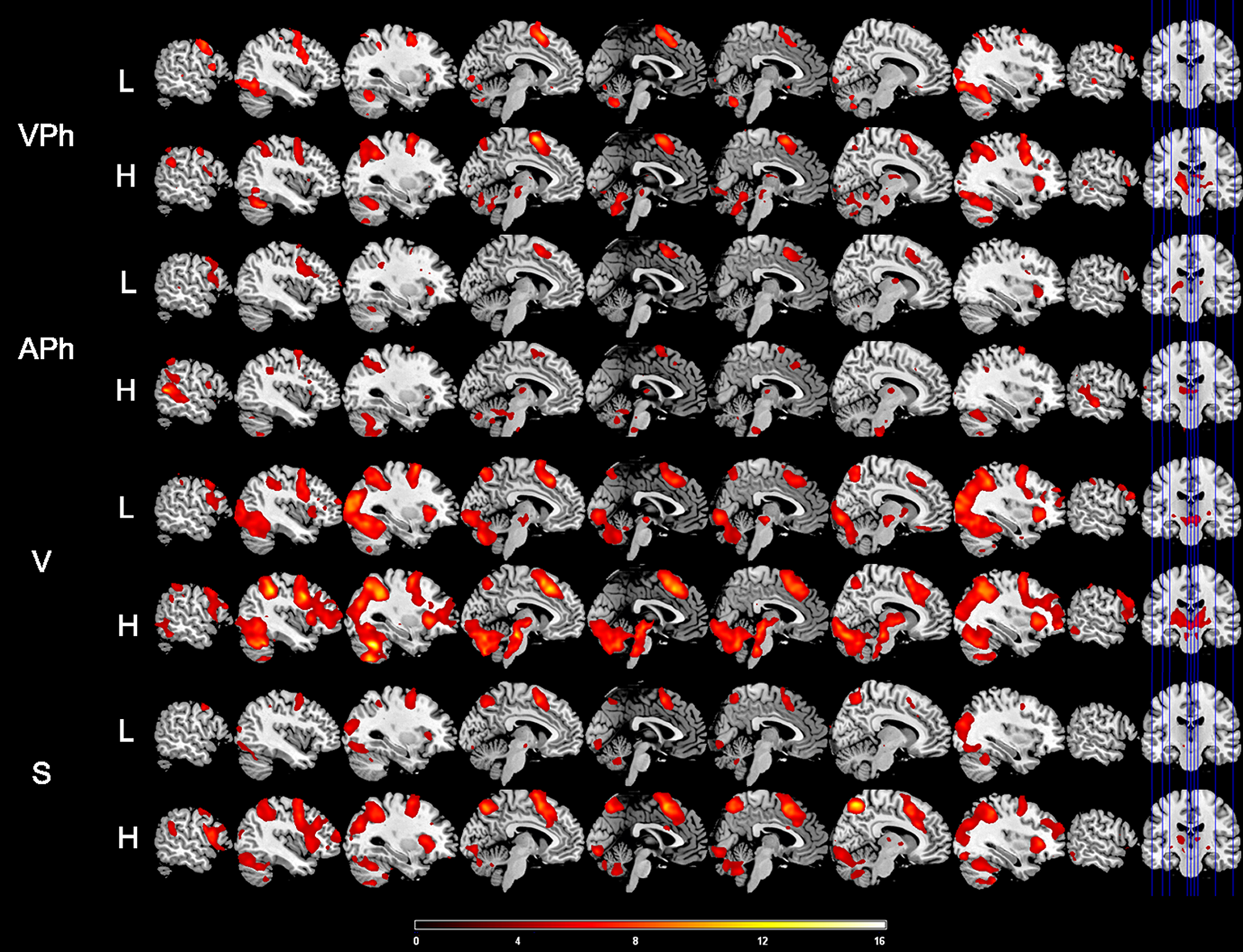

Supplement: S1 Fig — VPh: visual phonological version; APh: Auditory phonological version; V: visual; S: spatial; L: Low and H: High. The color scale represents T-statistic values. (p < 0.05 FWE cluster-wise corrected on voxels with p < 0.001). (TIF) [file pone.0131536.s001.tif]
