# Supplementary material for: Successful Working Memory Processes and Cerebellum in an Elderly Sample: A Neuropsychological and fMRI Study
Source: PLoS One. 2015 Jul 1;10(7):e0131536. doi: 10.1371/journal.pone.0131536 (PMC4488500; doi:10.1371/journal.pone.0131536)
Supplement: S1 File — (PDF) [file pone.0131536.s002.pdf]

Supporting Information

Introductory Notes

On the construction of behavioural tables: Significance threshold $p<0.05$. Significant comparisons have been marked using an asterisk.

On the construction of neuroimaging tables: Tables were created using the SPM8 Anatomy toolbox version 1.5 [1]. Anatomical areas reported are based in its V15 maps. Only significant clusters after a primary threshold of $p<0.001$ and a FWE cluster wise correction of $p<0.05$ are shown. All local maxima (more than 8 mm apart) located in these clusters are reported with their corresponding t-value and MNI coordinates (x,y,z; in mm). Cluster size and cluster-corrected p value are also reported.

Performance on the Neuropsychological Tests

In the neuropsychological assessment, the dependent variable (DV) of the articulatory suppression effect was the number of words recalled with and without suppression, the DV of the word length effect was the number of words recalled in each of the two lengths, the phonological similarities effect was determined by the number of words that participants stored in the similar and dissimilar conditions and Irrelevant sound effect was the number of words recalled in each of the two conditions (with Irrelevant sound and without Irrelevant sound effects).

Effective Connectivity Analysis

The PPI analysis applies the regression model $y1= ay0 + b (y0 \times u) +cu + X\beta$ [2]), where: y1 is the BOLD signal time series in a voxel, u is the task regressor (in our case the WM task), y0 is the time series of the seed, the PPI regressor $y0 \times u$ is a bilinear term formed by the element-by-element product

of the task regressor and the seed time series and Xβ is a constant term. The first principal component of the time series from the VOI within each area of interest was extracted. We were interested in areas that increased their connectivity with the seed areas during WM tasks; therefore we built t-maps showing the positive slope of the PPI regressor.

References

1. Eickhoff SB, Stephan KE, Mohlberg H, Grefkes C, Fink GR, Amunts K, et al. A new SPM toolbox for combining probabilistic cytoarchitectonic maps and functional imaging data. Neuroimage. 2005;25: 1325-1335.

2. Stephan KE. On the role of general system theory for functional neuroimaging. J Anat. 2004;205: 443-470.
