# Supplementary material for: Successful Working Memory Processes and Cerebellum in an Elderly Sample: A Neuropsychological and fMRI Study
Source: PLoS One. 2015 Jul 1;10(7):e0131536. doi: 10.1371/journal.pone.0131536 (PMC4488500; doi:10.1371/journal.pone.0131536)
Supplement: S1 Table — (PDF) [file pone.0131536.s003.pdf]

S1 Table. Data correct responses descriptive for each condition and load.

Condition	Load	*Task* Mean±SD	*Task*			*Control* Mean±SD	*Control task*		
			Condition (C)	*Load (L)*	*CXL*		*(C*	*L*	*CXL*
VPh	Low	91 ± 5.9	F/p			98 ± 2.0	F/p		
	High	88 ± 7.3	1.7/0.2	1.96/0.17	0.1/0.6	96 ± 4.7	5.3/0.003	3.9/0.06	5.1/0.003
APh	Low	91 ± 6.9	T test			96 ± 4.1	T test		
	High	89 ± 70				97 ± 3.5	VPh>V t= 3.2 p = 0.002		Low VPh>V t= 5.8 p = 0.0001
V	Low	91 ± 4.8				91 ± 4.8	APh>V t = 2.8 p = 0.007		Low APh>V t = 3.5 p = 0.002
	High	86 ± 7.4				96 ± 6.2	V<S t = -3.3 p 0.002		Low V<S t = -3.7 p 0.001
S	Low	89 ± 5.2				96 ± 4.5			
	High	88 ± 6.9				97 ± 3.5			
