# Supplementary material for: Successful Working Memory Processes and Cerebellum in an Elderly Sample: A Neuropsychological and fMRI Study
Source: PLoS One. 2015 Jul 1;10(7):e0131536. doi: 10.1371/journal.pone.0131536 (PMC4488500; doi:10.1371/journal.pone.0131536)
Supplement: S2 Table — (PDF) [file pone.0131536.s004.pdf]

S2A Table. Reaction time differences between conditions (correct responses only).

Condition	Load	*Task* Mean±SD	*Task*			*Control* Mean±SD	*Control task*		
			Condition (C)	*Load (L)*	*CXL*		*Condition (C)*	*Load (L)*	*CXL*
VPh	L	0.704 ± 0.122	F/p			0.632 ± 0.111	F/p		
	H	0.663 ± 0.133	36.2/0.001	1.24/0.2	0.09/0.7	0.606 ± 0.126	0.1/0.75	4.0/0.057	5.1/0.003
APh	L	0.434 ± 0.227	T test			0.438± 0.222	T test		
	H	0.456 ± 0.226	VPh>APh<br>t= 6.5<br>p = 0.0001			0.488 ± 0.185	VPh>APh<br>t= 5.1<br>p = 0.0001		Low<br>VPh>APh<br>t= 3.6<br>p = 0.002
V	L	0.758 ± 0.126	VPh<V<br>t = -4.5<br>p = 0.0001			0.618 ± 0.107	VPh>S<br>t = 3.9<br>p 0.0001		Low VPh>S<br>t= 3.0<br>p = 0.007
	H	0.733 ± 0.131	VPh<S<br>t = -3.3<br>p 0.0001			0.587 ± 0.122	APh<V<br>t= -4.5<br>p = 0.0001		Low APh<V<br>t= -3.4<br>p = 0.003
S	L	0.756 ± 0.138	APh<V<br>t= -9.1<br>p = 0.0001			0.572 ± 0.107	APh<S<br>t= -3.2<br>p = 0.002		Low APh<S<br>t= -2.4<br>p = 0.02
	H	0.739 ± 0.155	APh<S<br>t= -8.4<br>p = 0.0001			0.544 ± 0.140	V>S<br>t= 3.2<br>p = 0.02		Low V>S<br>t= 2.3<br>p = 0.03

Reaction time differences between stimulus conditions in the task condition (correct responses only). Corrected for multiple comparisons: Bonferroni method.

S2B Table. Reaction time differences between conditions.

Condition	Load	*Task* Mean±SD	*Task*			*Control* Mean±SD	*Control task*		
			Condition (C)	*Load (L)*	*CXL*		*Condition (C)*	*Load (L)*	*CXL*
									High<br>VPh>APh<br>t= 3.5<br>p = 0.002
									High<br>VPh>S<br>t= 2.6<br>p = 0.01
									High<br>APh<V<br>t= -2.9<br>p = 0.008
									High<br>APh<S<br>t= -2.0<br>p = 0.008
									High<br>V>S<br>t= 2.2<br>p = 0.03

Reaction time differences between stimulus conditions in the task condition (correct responses only). Corrected for multiple comparisons: Bonferroni method
