# Supplementary material for: Successful Working Memory Processes and Cerebellum in an Elderly Sample: A Neuropsychological and fMRI Study
Source: PLoS One. 2015 Jul 1;10(7):e0131536. doi: 10.1371/journal.pone.0131536 (PMC4488500; doi:10.1371/journal.pone.0131536)
Supplement: S3 Table — (PDF) [file pone.0131536.s005.pdf]

S3A Table. Differences between load activations analysis.

**Low vs. High load.**							**High vs. Low load.**						
Area	k	p	T	x	y	z	Area	k	p	T	x	y	z
Inferior occipital gyrus			5.90	35	-90	-6	Paracentral lobule			6.04	-3	-26	60
Middle occipital gyrus			5.63	27	-90	20				5.96	0	-23	68
	5116	0.0001	5.21	35	-87	12	SMA			5.52	9	-27	66
Lingual gyrus			4.86	6	-87	-6	Middle cingulate cortex	8161	0.0001	4.39	-2	-31	43
Cuneus			3.98	12	-99	9	Precuneus			4.35	3	-52	46
Rectal gyrus			5.75	6	29	-17				4.27	6	-58	43
Superior orbital gyrus			5.43	20	29	-18				3.73	-8	-63	45
			5.42	14	45	-21	Inferior parietal gyrus			5.41	-42	-57	44
Rectal gyrus	3195	0.0001	4.98	-8	18	-12				5.04	-48	-51	44
			4.46	5	50	-24	Middle temporal gyrus			4.87	-65	-41	-5
Superior orbital gyrus			3.76	-17	33	-23	Thalamus (Pulvinar Nuclei)	12459	0.0001	4.76	11	-28	7
			3.71	-15	32	-21	Inferior parietal gyrus			4.60	-53	-51	25
Inferior occipital gyrus			5.12	-30	-92	-9	Middle temporal gyrus			4.53	-65	-58	-1
Lingual gyrus	4223	0.0001	4.96	-20	-83	-15	Hippocampus			4.27	-14	-34	9
Fusiform gyrus			4.82	-26	-72	-11							

S3B Table. Differences between load activations analysis.

Low vs. High load.							High vs. Low load.						
Area	k	p	T	x	y	z	Area	k	p	T	x	y	z
							Middle frontal gyrus			5.19	-36	30	42
								4709	0.0001	4.33	-27	38	15
										4.07	-38	56	4
							Superior frontal gyrus			4.14	-24	53	0
							Middle frontal gyrus			4.70	33	36	24
							Caudate nucleus	2133	0.0001	4.22	21	2	23
										4.12	23	23	9
							Anterior cingulate Cortex			3.46	6	14	25
							Cerebellum VIIB lobule			4.55	-44	-56	-48
							Cerebellum VI lobule	2214	0.0001	4.26	17	-37	-20
							Fusiform gyrus			4.20	-33	-31	-27
