# Supplementary material for: Successful Working Memory Processes and Cerebellum in an Elderly Sample: A Neuropsychological and fMRI Study
Source: PLoS One. 2015 Jul 1;10(7):e0131536. doi: 10.1371/journal.pone.0131536 (PMC4488500; doi:10.1371/journal.pone.0131536)
Supplement: S4 Table — (PDF) [file pone.0131536.s006.pdf]

S4 Table. Differences between VPh and other conditions.

	APh						V						S					
	Area	k	T	x	Y	z	Area	k	T	x	y	z	Area	k	T	x	y	Z
VPh>	R Inferior occipital gyrus	790	5.48	42	-83	-11	L middle temporal gyrus	2138	6.09	-56	-68	26	R middle temporal gyrus	1288	4.65	65	-33	-8
	R inferior temporal gyrus		3.64	50	-69	-6			5.81	-50	-63	20			4.37	59	2	-20
	L Cerebellum Crus I lobe	1767	4.59	-44	-71	-26	L angular gyrus		5.71	-48	-74	42			4.34	57	-6	-15
	L inferior occipital gyrus		4.47	-44	-80	-5	R middle temporal gyrus	1400	5.86	56	-7	-18						
			4.13	-51	-74	0			5.36	62	-17	-12						
							L posterior cingulate cortex	2189	5.74	-5	-53	30						
							L superior temporal gyrus	1336	4.41	-54	-32	18						
									3.79	-62	-44	20						
