# Supplementary material for: Successful Working Memory Processes and Cerebellum in an Elderly Sample: A Neuropsychological and fMRI Study
Source: PLoS One. 2015 Jul 1;10(7):e0131536. doi: 10.1371/journal.pone.0131536 (PMC4488500; doi:10.1371/journal.pone.0131536)
Supplement: S5 Table — (PDF) [file pone.0131536.s007.pdf]

S5 Table. Differences between APh and other conditions.

	VPh						V						S					
	Area	k	T	x	y	z	Area	k	T	x	y	z	Area	k	T	x	y	Z
APh >	L middle temporal gyrus	2036	6.26	-56	-12	-5	L superior temporal gyrus	5987	7.49	-65	-26	9	L superior temporal gyrus	2941	6.69	-63	-17	7
	L superior temporal gyrus		5.46	-63	-21	7	L middle temporal gyrus		7.16	-59	-11	-8			5.10	-65	-33	12
									5.10	-60	2	-14	L middle temporal gyrus		5.89	-56	-14	-6
							R middle temporal gyrus	5686	6.39	63	-17	-12	R middle temporal gyrus	4375	5.73	63	-33	-2
									5.77	65	-33	1			4.78	63	-11	-15
							R heschis gyrus		5.86	44	-20	14			4.67	48	-20	-9

Differences between conditions activations analysis. L and R: Left and right laterality; FWE p: statistical significance and T-test score.
