# Supplementary material for: Successful Working Memory Processes and Cerebellum in an Elderly Sample: A Neuropsychological and fMRI Study
Source: PLoS One. 2015 Jul 1;10(7):e0131536. doi: 10.1371/journal.pone.0131536 (PMC4488500; doi:10.1371/journal.pone.0131536)
Supplement: S6 Table — (PDF) [file pone.0131536.s008.pdf]

S6A Table. Differences between V and other conditions.

	VPh						APh						S					
V>	Area	K	T	x	y	z	Area	k	t	x	y	z	Area	k	t	x	y	Z
	R middle occipital gyrus		13.98	35	-87	14	R middle occipital gyrus		14.08	33	-89	14	R inferior parietal lobule		10.19	32	-59	53
	L middle occipital gyrus	70896	13.22	-32	-87	20	L middle occipital gyrus	74598	13.98	-30	-80	24	L inferior temporal gyrus	44973	9.66	-48	-63	-8
	R cerebellum Crus I lobe		13.19	21	-81	-21			13.98	-30	-89	20	R lingual gyrus		9.38	14	-86	-8
	R inferior frontal gyrus		10.60	42	9	27	R inferior frontal gyrus		8.66	42	8	27	R inferior frontal gyrus		9.27	41	9	27
	R superior frontal gyrus	11596	7.31	29	0	51	R middle frontal gyrus	9081	7.18	29	-1	54		6934	6.08	53	29	29
	R insula lobe		7.55	33	21	0	R precentral gyrus		6.20	45	2	56	R middle frontal gyrus		6.55	45	38	15

S6B Table. Differences between V and other conditions.

	VPh						APh						S					
	Area	K	T	x	y	z	Area	k	t	x	y	z	Area	k	t	x	y	Z
	L inferior frontal gyrus		9.76	-42	5	30	L inferior frontal gyrus		8.08	-41	3	29	L inferior frontal gyrus	2064	7.83	-44	5	29
	L middle frontal gyrus	10788	7.76	-24	2	59	L middle frontal gyrus	5915	5.48	-24	14	71						
	L insula lobe		7.72	-30	21	4	L precentral gyrus		7.89	-27	-4	51						
V>	L superior medial frontal gyrus		8.36	-3	20	48	L SMA		7.14	-5	14	51						
	R anterior cingulate cortex	5133	5.91	9	24	32	L superior medial gyrus	3173	4.38	3	27	38						
	R SMA		3.97	3	15	71	R anterior cingulate cortex		4.22	9	24	32						
	L pallidum	1920	6.37	-17	-4	11			4.95	-36	54	30						
	R Pallidum		5.50	12	-1	4	L middle frontal gyrus	1383	4.85	-38	54	15						
	R Caudate N	952	5.16	17	-11	23			4.07	-33	42	14						
