# Supplementary material for: Successful Working Memory Processes and Cerebellum in an Elderly Sample: A Neuropsychological and fMRI Study
Source: PLoS One. 2015 Jul 1;10(7):e0131536. doi: 10.1371/journal.pone.0131536 (PMC4488500; doi:10.1371/journal.pone.0131536)
Supplement: S7 Table — (PDF) [file pone.0131536.s009.pdf]

S7A Table. Differences between S and other conditions.

	VPh						APh						S					
S>	Area	k	T	x	y	z	Area	k	T	x	y	z	Area	k	T	x	y	Z
	R precuneus	6763	8.12	15	-68	54	R precuneus	35931	9.04	14	-66	54	L middle temporal gyrus	2751	6.17	-48	-63	20
			7.38	9	-60	56	L precuneus		8.92	-14	-66	54	L superior temporal gyrus		4.68	-59	-44	23
	L precuneus		7.51	-14	-66	56	R middle occipital gyrus		8.82	38	-78	29	L angular gyrus		4.27	-48	-77	38
	R middle occipital gyrus	4678	7.76	38	-78	30	L middle frontal gyrus	3214	6.20	-23	0	56	L precuneus	2694	5.48	-14	-60	21
			6.93	32	-86	21			5.11	-23	8	68	R precuneus		4.68	12	-56	18
	R cuneus		4.03	14	-98	14	L precentrla gyrus		4.07	-45	-6	56	L posterior cingulate cortex		4.45	-2	-54	32
	L superior frontal gyrus	2264	6.37	-20	3	66												

S7B Table. Differences between S and other conditions.

	VPh						APh						S					
	Area	k	T	x	y	z	Area	k	T	x	y	z	Area	k	T	x	y	Z
S>	L middle occipital gyrus	2422	6.11	-39	-80	17												
	L fusiform gyrus	1231	5.52	-26	-66	-11												
	L fusiform gyrus		3.79	-32	-48	-12												
	R lingual gyrus	947	5.07	23	-72	-9												
	R fusiform		3.91	27	-56	-8												
	L insula lobe		4.93	-33	15	11												
	L inferior frontal gyrus	1040	4.29	-45	30	-5												
	R anterior cingulate cortex	1496	4.32	5	30	30												
	L anterior cingulate cortex		4.22	-5	24	32												
