# Supplementary material for: Successful Working Memory Processes and Cerebellum in an Elderly Sample: A Neuropsychological and fMRI Study
Source: PLoS One. 2015 Jul 1;10(7):e0131536. doi: 10.1371/journal.pone.0131536 (PMC4488500; doi:10.1371/journal.pone.0131536)
Supplement: S8 Table — (PDF) [file pone.0131536.s010.pdf]

S8 Table. PPI for low and high loads visual.

low load visual							
Seed	Connecting area	K	FWE p	T	x	y	z
R Thalamus (Ventral lateral nucleus)	L Inferior occipital gyrus	1583	0.0001	5.71	-47	-71	-11
	L Cerebellum Crus I lobe			5.18	-27	-77	-20
L Cerebellum VI lobe	L Cerebellum Crus I lobe	1106	0.001	6.38	-26	-78	-21
	L Inferior occipital gyrus			5.24	-41	-66	-6
High load visual							
Seed	Connecting area	k	FWE p	T	x	y	z
R Thalamus (Ventral lateral nucleus)	L Paracentral	982	0.008	6.77	-5	-41	78
	R Paracentral			5.33	3	-29	74
R Cerebellum VIIIA and Crus I lobules	L Lingual gyrus	1724	0.0001	6.75	-12	-87	-12
	L Fusiform gyrus			5.38	-38	-80	-14

L and R: Left and right laterality; FWE p: statistical significance and T-test score.
