# Supplementary material for: Successful Working Memory Processes and Cerebellum in an Elderly Sample: A Neuropsychological and fMRI Study
Source: PLoS One. 2015 Jul 1;10(7):e0131536. doi: 10.1371/journal.pone.0131536 (PMC4488500; doi:10.1371/journal.pone.0131536)
Supplement: S9 Table — (PDF) [file pone.0131536.s011.pdf]

S9 Table. PPI for high load visual phonological and auditory phonological

high load visual phonological

Seed	Connecting area	k	FWE p	T	x	y	z
R Cerebellum VI lobe	L Precentral gyrus	513	0.03	4.47	-41	-22	64
				4.32	-33	-22	58
				4.11	-50	-6	54
R Cerebellum VIIIA and Crus I lobules	R SMA	528	0.02	5.35	5	14	62
	L SMA			4.25	2	11	51

high load auditory phonological

Seed	Connecting area	k	FWE p	T	x	y	z
R Thalamus (Ventral lateral nucleus)	L Superior frontal	2870	0.0001	7.47	-32	-1	69
	L Precentral			7.02	-35	-14	69
	L Postcentral gyrus			5.13	-57	-17	30
	L preSMA	1004	0.006	4.93	0	14	53
	R preSMA			4.82	3	-1	57
R Pallidum	L Precentral gyrus			4.64	-53	-4	45
	L Postcentral gyrus			4.54	-50	-9	54
R Cerebellum VIIIA and Crus I lobules	R Superior medial frontal gyrus	4446	0.0001	5.15	5	33	54
	L Insula lobe	837	0.01	6.22	-39	12	-2
	L DLPFC	605	0.04	5.18	-29	45	35
	L Superior frontal gyrus			4.34	-17	60	24
	R DLPFC	746	0.01	5.16	29	47	15

L and R: Left and right laterality; FWE p: statistical significance and T-test score.
