# Supplementary material for: Successful Working Memory Processes and Cerebellum in an Elderly Sample: A Neuropsychological and fMRI Study
Source: PLoS One. 2015 Jul 1;10(7):e0131536. doi: 10.1371/journal.pone.0131536 (PMC4488500; doi:10.1371/journal.pone.0131536)
Supplement: S10 Table — (PDF) [file pone.0131536.s012.pdf]

S10 Table. PPI for high load spatial.

Seed	Connecting area	k	FWE p	T	x	y	Z
R Thalamus (Ventral lateral nucleus)	R Middle occipital gyrus	754	0.0228	5.33	32	-83	27
Vermal lobule IX	R Cerebellum VI lobe	885	0.0239	5.53	23	-66	-24
R Cerebellum VIIIA and Crus I lobules	L Cerebellum VIIA and Crus I lobes	1259	0.0032	5.84	-5	-80	-27
	R Cerebellum VI lobe			5.04	11	-75	-17

L and R: Left and right laterality; FWE p: statistical significance and T-test score.
